# Impaired thymic iNKT cell differentiation at early precursor stage in murine haploidentical bone marrow transplantation with GvHD

**DOI:** 10.3389/fimmu.2023.1203614

**Published:** 2023-08-03

**Authors:** Weijia Zhao, Yujia Wang, Xinwei Zhang, Jie Hao, Kunshan Zhang, Xiaojun Huang, Yingjun Chang, Hounan Wu, Rong Jin, Qing Ge

**Affiliations:** ^1^ Department of Immunology, School of Basic Medical Sciences, Peking University, National Health Commission (NHC) Key Laboratory of Medical Immunology (Peking University), Beijing, China; ^2^ Central Lab, Tongji Hospital, School of Medicine, Tongji University, Shanghai, China; ^3^ Beijing Key Laboratory of Hematopoietic Stem Cell Transplantation, Peking University People’s Hospital & Institute of Hematology, Beijing, China; ^4^ Peking University Medical and Health Analytical Center, Peking University, Beijing, China; ^5^ Department of Integration of Chinese and Western Medicine, School of Basic Medical Sciences, Peking University, Beijing, China

**Keywords:** invariant natural killer T cells, haploidentical bone marrow transplantation, thymus, NKT1 precursor, graft-versus host disease

## Abstract

**Introduction:**

Early recovery of donor-derived invariant natural killer T (iNKT) cells are associated with reduced risk of graft-versus-host disease (GvHD) and overall survival. Patients with severe GvHD, however, had much slower iNKT cell reconstitution relative to conventional T cells.

**Methods:**

To characterize the delay of iNKT cell reconstitution and explore its possible causes, we used a haploidentical bone marrow transplantation (haplo-BMT) mouse model with GvHD. We found the delayed recovery of thymic and peripheral iNKT cell numbers with markedly decreased thymic NKT1 subset in GvHD mice. The defective generation of thymic iNKT precursors with egress capability contributed to the reduced peripheral iNKT cells in GvHD mice. We further identified intermediate NK1.1^-^ NKT1 precursor subpopulations under steady-state conditions and found that the differentiation of these subpopulations was impaired in the thymi of GvHD mice. Detailed characterization of iNKT precursors and thymic microenvironment showed a close association of elevated TCR/co-stimulatory signaling provided by double positive thymocytes and macrophages with defective down-regulation of proliferation, metabolism, and NKT2 signature in iNKT precursor cells. Correspondingly, NKT2 but not NKT1 differentiation was favored in GvHD mice.

**Discussion:**

These data underline the important roles of TCR and co-stimulatory signaling in the differentiation of thymic iNKT subsets under transplantation conditions.

## Introduction

Invariant natural killer T (iNKT) cells are a subset of unconventional αβ T cells that express a semi-invariant T cell receptor (TCR), recognize glycolipid antigens presented on non-polymorphic MHC class Ib molecule CD1d, and regulate immune responses with both innate and adaptive characteristics (1). The important roles of iNKT cells are well appreciated in allogeneic hematopoietic stem cell transplantation (allo-HSCT) and combined bone marrow (BM) and organ transplantation. iNKT cells from recipients, donors, or third-party can reduce the risk of graft-versus-host disease (GvHD) while retaining the graft-versus-leukemia/lymphoma (GvL) effect (2–7). Human CD4^-^ NKT1 cells, CD4^+^ IL-4-producing iNKT cells, or murine CD4^+^ NKT2 cells are effective in this respect (8–10). In addition, the effector cytokines generated by thymic iNKT cells also contribute to the regulation of conventional T cell development and possibly reconstitution (1, 11, 12). Thus, early recovery of donor-derived iNKT cells and high iNKT/T ratios have been reported to be significantly associated with reduced non-relapse mortality and reduced risk of acute GvHD (3, 13–16). In patients with severe GvHD, however, the reconstitution of iNKT cells is a much slower process when compared to conventional T cells, irrespective of umbilical cord-derived or BM-derived donor transplantation (2, 16–19). When iNKT (human) subsets were analyzed, the recovery of CD4^-^ NKT1 cells was much slower than CD4^+^ iNKT cells, reaching a massive expansion 4-6 years post-HSCT in pediatric patients post-HSCT (16). The reasons for such a severe delay in NKT1 reconstitution remain unclear.

Thymus is the primary site of iNKT cell development. After positive selection by CD1d-expressing CD4^+^CD8^+^ double positive (DP) thymocytes, murine thymic Vα14i (Vα14-Jα18)-expressing cells quickly acquire iNKT progenitor cell fate (stage 0, ST0, or NKT0) and upregulate CD69, CD24, and transcription factor Egr2 (20, 21). Co-stimulatory signals from SLAM-SAP promote PLZF (*Zbtb16*) expression and signals from CD28 promote survival and proliferation of PLZF-expressing progenitors (20–23). The post-selected iNKT cells subsequently become functionally distinct effector subsets but their differentiation journey in the thymus is not completely understood (24, 25). CD44 and NK1.1 were used to define simplified developmental stages, including ST1 (CD24^-^CD44^-^), ST2 (CD24^-^CD44^+^NK1.1^-^), and ST3 (CD24^-^CD44^+^NK1.1^+^) (26–28). This model was substantially revised later by a combination of CCR7 and transcription factors (29, 30). Thus, CD24^+^ ST0 cells were followed by CD24^-^CCR7^+^PLZF^hi^ intermediate progenitor (NKTp) cells that can either emigrate to the periphery and mature on-site or give rise to 3 different CD44^hi^ effector iNKT subsets within the thymus, NKT1 (PLZF^lo^T-bet^+^), NKT2 (PLZF^hi^GATA3^+^), and NKT17 (PLZF^int^RORγt^+^). The developmental intermediate subsets between NKTp and these differentiated functional subsets were not fully defined. Recently, single-cell RNA sequencing (scRNA-seq) analysis, *in vitro* differentiation experiments, and an inducible Vα14i TCR rearrangement system suggest that thymic PLZF^hi^ NKTp and NKT2-like subsets represent developmental intermediate stages that contain precursors of NK1.1^+^ NKT1 as well as CD138^+^ NKT17 subsets (20, 21, 31–35). Three subpopulations were also defined within NK1.1^+^ NKT1 or ST3 cells based on their expression intensity of NK1.1 and Sca-1 (31). However, ST3 cells constitute more than 60% of thymic iNKT cells in C57BL/6 mice. The inclusion of this subset in scRNA-seq precludes a comprehensive analysis of NK1.1^-^CD24^-^ intermediate precursor cells. It has been shown that CD44^hi^NK1.1^-^ thymocytes could give rise to NK1.1^+^ iNKT cells 7 days after intrathymic injection while IL-4-producing iNKT cells that also fall within CD44^hi^NK1.1^-^ stage failed to become T-bet^+^ NKT1 cells (29, 36). Thus, an enrichment of ST2 and even ST1 cells and further identification of NKT1 precursor subpopulations within these stages are warranted.

To investigate the possible reasons for the severe delay of NKT1 reconstitution in BM transplantation (BMT) with GvHD, we combined flow cytometry and scRNA-seq analysis to characterize thymic and peripheral iNKT cells in a haploidentical BMT (haplo-BMT) mouse model. Thymic ST1 and ST2 iNKT cells were purified to examine the presence of NK1.1^-^ NKT1 precursors, whether and how they are affected by GvHD. We found reduced numbers of NKTp cells in the thymus and recent thymic iNKT emigrants in the periphery of GvHD mice, likely contributing to the delayed reconstitution of peripheral iNKT cell numbers after haplo-BMT. The mice with GvHD further showed impaired differentiation of the NKT1 effector subset at the early precursor stage, leading to a markedly decreased NKT1/NKT2 ratio in GvHD mice. The thymic microenvironment that contributes to NKT1 changes was explored.

## Results

### Impaired iNKT cell reconstitution in haplo-BMT mice with GvHD

**Figure 1:**
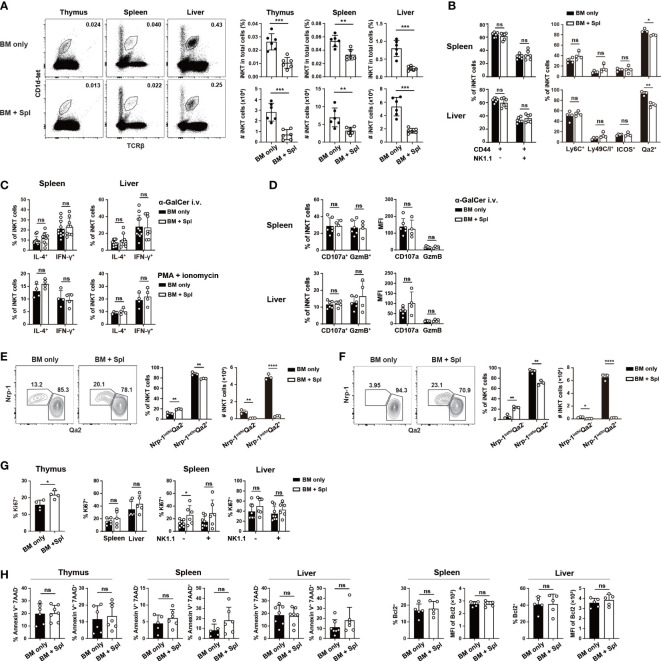
Haplo-BMT mice with GvHD had reduced thymic and peripheral iNKT cells. CD3^+^ T cell-depleted BM cells (5 x 10 (6)) from C57BL/6 mice were adoptively transferred into lethally irradiated F1 (C57BL/6 x BALB/c) mice. Some of the recipients were also injected with C57BL/6-derived 2 x 10 (6) splenic cells and were assigned as BM + Spl or GvHD. The recipients that did not receive splenic cells were assigned as BM only. All the recipients were analyzed at 9 weeks post haplo-BMT. **(A)** Flow cytometry analysis of iNKT cells (stained with PBS57-loaded CD1d tetramer (CD1d-tet) and TCRβ) in the thymus, spleen, and liver of Haplo-BMT mice with BM only and BM + Spl. The percentage (upper panels) and number (lower panels) of iNKT cells were calculated on the right. **(B)** Flow cytometry comparison of the expression of maturation markers (CD44, NK1.1, Ly6C, Ly49C/I, ICOS, Qa2) in iNKT cells in the spleen and liver. The percentages of CD44^+^NK1.1^-^, CD44^+^NK1.1^+^ cells, Ly6C^+^, Ly49C/I^+^, ICOS^+^, Qa2^+^ cells were compared. **(C, D)** Flow cytometry analysis of IFN-γ^+^, IL-4^+^ CD107a^+^, Granzyme B (GzmB)^+^ iNKT cells in the spleen and liver upon stimulation with α-GalCer *in vivo* or PMA/ionomycin *in vitro*. Two hours after intravenous injection of α-GalCer, the cells from the spleen and liver of haplo-BMT mice with BM only and BM + Spl were harvested (38), cultured in the presence of Brefeldin A (3 μg/ml) for 3 hours, and stained for intracellular cytokines and cytotoxicity-related molecules. The cells were also harvested from unchallenged mice and stimulated *in vitro* by PMA and ionomycin for 4 hours, and stained for intracellular cytokines. **(E, F)** Flow cytometry analysis of Nrp1^+^Qa2^int^, Nrp1^-^Qa2^hi^ iNKT cells obtained from the spleen **(E)** and liver **(F)**. **(G)** Flow cytometry analysis of Ki67^+^ iNKT cells obtained from the thymus, spleen, and liver of haplo-BMT mice with BM only or BM + Spl. **(H)** Flow cytometry analysis of Annexin V^+^7-AAD^-^, Annexin V^+^7-AAD^+^ and Bcl2^+^ iNKT cells in the thymus, spleen, and liver of haplo-BMT mice with BM only or BM + Spl. Data are representative of at least 3 independent experiments. Student’s t test was used for statistical analysis. *P < 0.05, **P < 0.01, ***P < 0.001, ****P < 0.0001, ns, not significant.

To examine the reconstitution of iNKT cells in haplo-BMT with and without GvHD, CD3^+^ T cell-depleted BM cells (5x10 (6)) from C57BL/6 mice were adoptively transferred into lethally irradiated F1 (C57BL/6 x BALB/c) mice with or without 2x10 (6) B6-derived splenic cells (BM + Spl (GvHD) versus BM only) (**Figures S1A–C**), (**37**). At 9- and 13-week post-BMT, the cell numbers, frequencies of iNKT cells (TCRβ^+^PBS57:CD1d-tetramer (CD1d-tet)^+^), and iNKT/T ratios in the thymus, spleen, and liver were significantly lower in mice with GvHD than those with BM only (**Figures 1A**; **S1D**), agreeing well with the previous results of impaired iNKT cell reconstitution in allo-HSCT patients with GvHD (2, 16–19). Except for a slight decrease in Qa2 expression, the maturation of peripheral iNKT cells, measured by the expression of surface markers (CD44, NK1.1, Ly6C, Ly49C/I) and cytotoxicity-related molecules (CD107a, Granzyme B), production of cytokines (IL-4 and IFN-γ), exhibited no substantial differences between mice with and without GvHD (**Figures 1B–D**; **S1E, F**). Nrp1 is expressed in recent thymic iNKT emigrants but not mature iNKT cells (39). We found an increased relative percentage of Nrp1^int/hi^Qa2^-^ iNKT cells but a substantially decreased number of both Nrp1^int/hi^Qa2^-^ and Nrp1^int/lo^Qa2^+^ iNKT cells in the spleen and liver of GvHD mice (**Figures 1E, F**). The comparison of Ki67^+^ iNKT cells showed enhanced proliferation in the thymus but not the periphery of GvHD mice (**Figure 1G**). No difference was found in the levels of iNKT cell apoptosis/survival (AnnexinV^+^7-AAD^-^, AnnexinV^+^7-AAD^+^, or Bcl2^+^ cells) between the two groups (**Figure 1H**). As more than 95% of iNKT cells in mice with GvHD were of donor origin (CD45.1^+^) (**Figure S1G**), the reduced iNKT cells in the thymus and decreased recent thymic iNKT emigrants in the periphery suggest that *de novo* generation of thymic iNKT cells is impaired in GvHD mice.

### Impaired thymic NKTp and NKT1 development in haplo-BMT mice with GvHD

**Figure 2:**
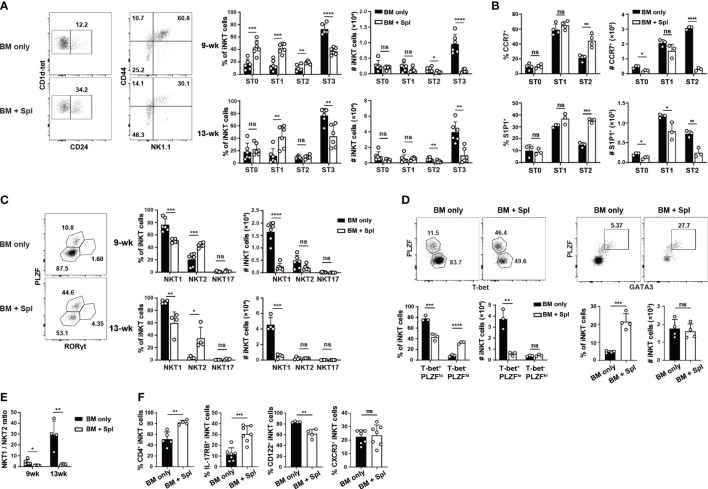
Haplo-BMT mice with GvHD had reduced NKT1 cells in the thymus. **(A)** Flow cytometry analysis of thymic iNKT cells at various stages in mice with BM only and those with BM + Spl at 9 weeks after haplo-BMT. ST0, CD24^+^CD44^lo^, ST1, CD24^-^CD44^lo^, ST2, CD44^hi^NK1.1^-^, ST3, CD44^hi^NK1.1^+^. **(B)** Flow cytometry comparison of emigration-related molecules CCR7 and S1P1 in iNKT cells at various stages 9 weeks after haplo-BMT. **(C)** Flow cytometry analysis of thymic effector iNKT subsets in haplo-BMT mice after 9 and 13 weeks of transplantation. NKT2 subset, PLZF^hi^RORγt^-^, NKT17 subset, PLZF^int^RORγt^+^, NKT1 subset, PLZF^lo^RORγt^-^. **(D)** Flow cytometry analysis of thymic iNKT subsets at 9 weeks post BMT. Left panel, T-bet^+^PLZF^lo^ as NKT1 subset and T-bet^-^PLZF^hi^ as a mixture of NKT2 and NKT17 subsets. Right panel, GATA3^+^PLZF^hi^ as NKT2 cells. **(E)** Comparison of thymic NKT1/NKT2 ratios between the groups of BM only and BM + Spl at 9 and 13 weeks post BMT. **(F)** Flow cytometry analysis of the percentages of CD4^+^, IL-17RB^+^, CD122^+^, and CXCR3^+^ iNKT cells in the thymi at 9 weeks after haplo-BMT. *P < 0.05, **P < 0.01, ***P < 0.001, ****P < 0.0001, ns, not significant.

To determine the underlying cause of iNKT cell defects in the thymus, we first examined which iNKT cell stage or effector subset is affected. Compared to BMT mice with BM only, those with GvHD showed a marked loss of ST2/ST3 cells and a relative accumulation of ST0/ST1 cells (**Figures 2A**; **S1H**). We further observed a significant decrease in the cell number of the thymic NKTp subset (CCR7^+^ or S1P1^+^ cells) at various stages (**Figure 2B**), agreeing well with the reduced Nrp1^+^ thymic iNKT emigrants in the periphery of GvHD mice. We also stained the cells with antibodies specific for transcription factors and surface markers related to iNKT effector subsets. Consistent with ST3 cell reduction, the mice with GvHD had a marked reduction of PLZF^lo^RORγt^-^ or PLZF^lo^T-bet^+^ NKT1 cells (**Figures 2C, D**). The percentages of PLZF^hi^RORγt^-^ or PLZF^hi^GATA3^+^ NKT2 cells were increased while the cell numbers were similar in GvHD mice relative to the controls, leading to a substantial decrease in the ratio of NKT1/NKT2 cells, in particular at 13-week after haplo-BMT (**Figures 2C–E**). No significant difference was found in PLZF^int^RORγt^+^ NKT17 cells in GvHD mice (**Figure 2C**). The expression of surface markers of NKT2 (CD4, IL-17RB) and NKT1 (CD122, CXCR3) cells showed a similar pattern of changes in GvHD mice (**Figure 2F**). Together, these results indicate a profound impairment of NKTp and NKT1 generation in the thymus of haplo-BMT mice with GvHD.

### Identification of NK1.1^-^ NKT1 precursors in the thymus of C57BL/6 mice

**Figure 3:**
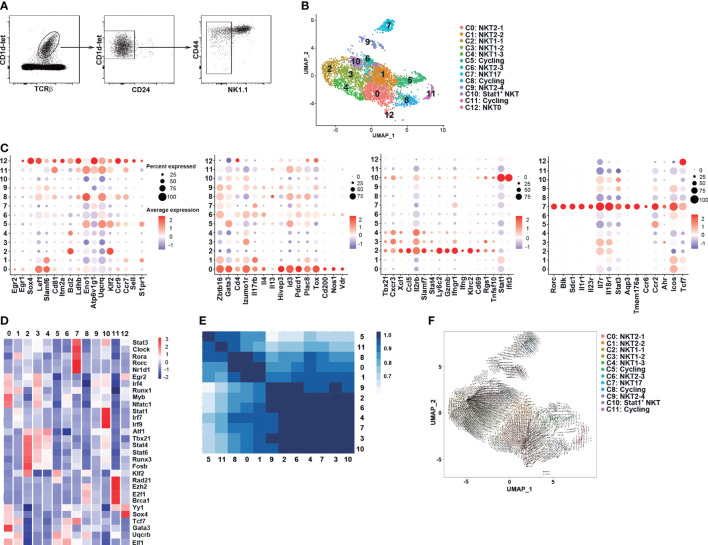
scRNA-seq identified NK1.1^-^ NKT1 precursor clusters in C57BL/6 thymi at steady state. **(A)** CD1d-tet^+^TCRβ^+^CD24^-^NK1.1^-^ thymocytes were sorted from the thymus of 8-week old C57BL/6 mice (n = 8) and were sent for scRNA-seq. **(B)** UMAP visualization of iNKT cell clusters. **(C)** Dot plots showing the expression of selected gens (x axis) by cluster (y axis). The Dot size represents the fraction of cells in the cluster that express the gene; color indicates the mean expression (logTPX) in expressing cells relative to other clusters. **(D)** Heatmap of cell type specific TFs in each cluster, with mean AUC scores being shown. **(E)** Calculation of Pearson correlation between clusters. **(F)** RNA velocities are visualized on the pre-defined UMAP plot from Seurat coordinates of clusters C0-11. The averaged gene expression profiles of cells within a neighborhood are represented by arrows. The speed of development is represented by length of arrow.

Thymic NKT1 cells have been reported to derive from NKTp and NKT2-like cells at developmental stages of ST1 and ST2. The intermediate NKT1 precursors, however, were more clearly defined within ST3 cells (31). As GvHD thymi showed a substantial decrease in NKT1/ST3 cells but not NKT2 and NKT17 cells, we determined to define early NKT1 precursors at ST1 or ST2 and investigate whether these precursors were affected in GvHD mice. We first explored NKT1 precursors in post-selected TCRβ^+^CD1d-tet^+^CD24^-^NK1.1^-^ iNKT cells (ST1 and ST2) in C57BL/6 mice without BMT by scRNA-seq (**Figure 3A**). A total of 4491 cells passed the quality control and 13 clusters were identified by unsupervised clustering analysis based on top 20 principal components (**Figures 3B**; **S2A, B**). In line with the enrichment of CD24^-^ iNKT cells, the transcript of *Cd24a* was only found in a few scattered cells in cluster C12 and cycling cluster C11 (**Figures S2C, D**). The cells in cluster C12 were assigned as NKT0 as they displayed enriched expression of genes associated with post-selected ST0 cells, including lymphocyte activation (*Egr1, Sox4*), metabolism (*Ldhb, Uqcrq*), and trafficking (*Ccr7*) (**Figure 3C**) (40–42). High activity of transcription factors Yy1 and Sox4 (30, 40–44) were also found in C12 by transcription factor regulatory network analysis (**Figure 3D**). The cells in clusters C0, C1, C6, and C9 were categorized as NKT2 or NKT2-like cells as they displayed NKT2 signature with cluster C0 having the highest level of NKT2 related genes (*Zbtb16, Gata3, Pdcd1, Tox, Id3, Il4 Il17rb, Slamf6*, and *Izumol1r*) and highest activity of transcription factors *Gata3* and *Myb* (**Figures 3C, D**). We also assigned the clusters C2, C3, and C4 as NKT1 for their high expression of NKT1 signature (*Tbx21, Cxcr3, Xcl1, Il2rb, Slamf7*, killer cell lectin type receptors, cytotoxicity-related genes) and high activity of transcription factors *Tbx21* and *Stat4* (**Figures 3C, D**). Among these three clusters, C2 showed the highest expression of NKT1 markers as well as cell retention marker *Cd69*, suggesting that C2 cells are more closely related to NK1.1^+^CD69^+^ mature NKT1 cells.

Based on the published signature genes of various iNKT subsets (31–33) and transcription factor regulatory network analysis, other clusters were classified as cycling clusters (C5, C8, and C11), NKT17 (C7), and Stat1^+^ NKT (**Figures 3C, D**; **S2C, D**). Notably, relatively high levels of *Gata3, Id3, Pdcd1*, and *Il13* transcription were found in cycling cell clusters (**Figure 3C**), supporting the previous finding of high proliferation capacity in NKT2 cells (45). The Stat1^+^ NKT cluster showed differential expression of NKT1 (*Tbx21, Il2rb, Cxcr3*) and NKT2 (*Gata3*, *Izumo1r*) signature genes, and retention marker *Cd69* (**Figure 3C**), suggesting that this cluster may contain cells at the developmental intermediate stage that have lost some trafficking capability. We further found that C1, C12, cycling clusters, and to a lesser extent, C0, expressed NKTp marker *Ccr7* while C1, C5, and C12 showed a high level of *S1pr1*, suggesting that these clusters are enriched with NKTp cells.

We applied Pearson correlation to assess the similarities between clusters. As shown in **Figure 3E**, NKT2 clusters (C0 and C1) formed a distinct group and showed a high degree of similarity with cycling clusters (C5, C8, C11). NKT1 clusters (C2, C3, and C4), NKT2 clusters (C6, C9), NKT17 cluster (C7), and Stat1^+^ NKT cluster (C10) formed another group with a higher degree of similarity with cluster C1. We further performed RNA velocity analysis (**Figure 3F**) and found that clusters C2 and C0 represented well-differentiated NKT1 and NKT2 effector subpopulations, respectively. The NKT1 clusters (C3, C4), NKT2 clusters (C6, C9), and Stat1^+^ NKT cluster (C10) all had precursor cells for NKT1 cluster C2, suggesting that these clusters may represent distinct but intermediate precursor stages during NKT1 differentiation (**Figure 3F**). Notably, a small fraction of cells in NKT2 cluster C1 appeared to be precursors for C3, C6, and C10 while the rest of the cells had precursors for NKT2 cluster C0 (**Figure 3F**). Collectively, the scRNA-seq data suggest that C1 may be a critical *Ccr7* ^+^ NKTp cluster that can either emigrate from the thymus (*S1pr1*) or differentiate into NKT1 (C2) and NKT2 (C0) effector clusters within the thymus. C6, C9, C10, C3, and C4 may represent intermediate clusters gradually downregulating the NKT2 signature while upregulating the NKT1 signature.

### Reduced NKT1 clusters with upregulated NKT2 signature and metabolism in the thymus of haplo-BMT mice with GVHD

**Figure 4:**
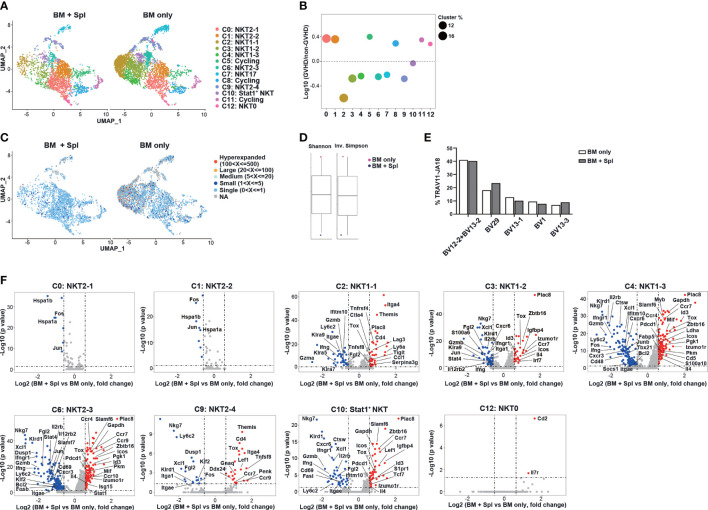
Haplo-BMT mice with GvHD had reduced thymic NKT1 precursor clusters with upregulated NKT2 signature. **(A)** UMAP visualization of scRNA-seq samples of thymic CD1d-tet^+^TCRβ^+^CD24^-^NK1.1^-^ iNKT cell clusters in haplo-BMT mice with BM only (n = 12) and BM + Spl (n = 15). **(B)** Comparison of iNKT cell cluster frequencies. The y axis shows Log10 of the ratio of the frequencies of the indicated clusters. The dot size represents the frequency of each cluster within its own sample. The x axis and dot color show indicated clusters. **(C)** UMAP plots of individual sc*Tcr*-seq samples with cells colored according to the size of their TCR clonotypes. **(D)** Comparison of the clonal diversity of sc*Tcr*-seq samples analyzed by Shannon diversity index (left) and Inversed Simpson index (right). **(E)** Comparison of the frequencies of TRBV usage in iNKT cells with TRAV11-JA18. A total of 1992 cells in BM only and 1505 cells in BM + Spl with TRAV11-JA18 were analyzed. **(F)** Volcano plots showing differentially expressed genes in various iNKT clusters obtained from the comparison of BM only and BM + Spleen samples. The dot lines show 1.5-fold cutoff.

We next analyzed scRNA-seq data of thymic CD24^-^NK1.1^-^ iNKT cells obtained from haplo-BMT mice with and without GvHD (**Figure 4A**). Compared to those with BM only, the mice with GvHD had markedly reduced proportions of cells in NKT1 cluster C2 and intermediate precursor clusters C3, C4, C6, C9 (**Figures 4A, B**). The percentages of NKT2 (C0, C1), NKT0 (C12), and cycling clusters (C5, C8, C11), however, were increased in mice with GvHD. We also found that mice without GvHD had enrichment of TCR clonal expansion in NKT1 and their intermediate precursor clusters (clusters C2, C3, C4, and C6) while those with GvHD only had small or single-cell clones (**Figure 4C**). Accordingly, higher clonal diversity was found in mice without GvHD (**Figure 4D**). However, the comparison of TCR Vβ usage and CDR3 length of Vβ chain showed no difference between the two groups (**Figures 4E**, **S2E**).

We further compared the transcriptome of each cluster and found that NKT1 and precursor clusters (C2, C3, C4, C6, C9, C10) in GvHD thymi had upregulation of NKT2-signature genes (*Zbtb16, Pdcd1, Izumo1r, Tox, Il4*), chemokine receptor (*Ccr7*), interferon-responsive genes (*Irf7, Stat1*), and down-regulation of NKT1 signature genes (*Tbx21*, *Gzmb, Nkg7, Stat4, Xcl1*) (**Figure 4F**). Increased expression of genes related to glucose metabolisms (*Pkm, Pgk1*) was also observed in NKT1 precursor clusters C4 and C6 in GvHD mice. GSEA analysis of clusters C3, C4, and C6 further showed that iNKT cells in GvHD mice had down-regulation of NK-mediated cytotoxicity, inflammatory response, and upregulation of oxidative phosphorylation, citric acid TCA cycle & respiratory electron transport, metabolism of amino acids & derivatives (**Figure S2F**).

### Flow cytometric characterization of NK1.1^-^ NKT1 precursor subpopulations in mice with and without Haplo-BMT

**Figure 5:**
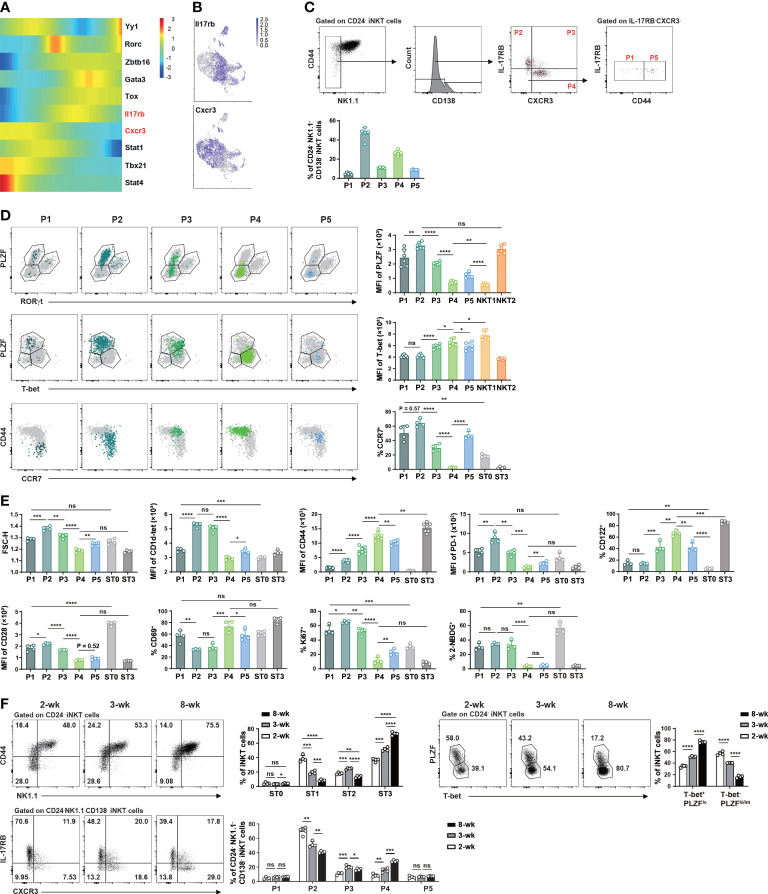
Flow cytometry identified NK1.1^-^ NKT1 precursor subpopulations in the thymus of C57BL/6 mice at steady state. **(A)** Heatmap showing differentially expressed genes related to various iNKT clusters across the pseudotime using Monocle2. Color key from blue to red indicates relative expression levels from low to high. **(B)** UMAP plots as in **Figure 3A** with color code displaying the expression of *Il17rb* and *Cxcr3* (gray for low and purple for high expression). **(C)** Gating strategies to identify subpopulations within CD24^-^NK1.1^-^CD138^-^ iNKT cells and comparison of the proportions of various subpopulations. P1, IL-17RB^-^CXCR3^-^CD44^lo^, P2, IL-17RB^+^CXCR3^-^CD44^lo/int^, P3, IL-17RB^+^CXCR4^+^CD44^int/hi^, P4, IL-17RB^-^CXCR3^+^CD44^hi^, P5, IL-17RB^-^CXCR3^-^CD44^int/hi^. **(D)** Flow cytometry comparison of the expression of transcription factors (PLZF, RORγt, T-bet) and surface molecules (CD44, CCR7) in these subpopulations. **(E)** Flow cytometry analysis of cell size, cell proliferation (Ki67), glucose uptake (2-NBDG), and expression of selected surface markers in iNKT subpopulations. **(F)** Flow cytometry analysis of the frequency changes of iNKT cells at various stages (upper left panel), in NKT1 (T-bet^+^PLZF^lo^)/non-NKT1 subsets (upper right panel), and in various NK1.1^-^ subpopulations (lower panel) in C57BL/6 mice of 2-, 3-, and 8-week old. *P < 0.05, **P < 0.01, ***P < 0.001, ****P < 0.0001, ns, not significant.

To assess whether NKT1 precursor subpopulations within ST1 and ST2 could be identified by flow cytometry, we first analyzed scRNA-seq data (**Figure 3B**) again to create a pseudo-time ordering of iNKT cell transcriptomes. As shown in **Figure 5A**, *Il17rb* and *Cxcr3* were upregulated along with *Gata3* and *Tbx21*, respectively. The clusters at intermediate developmental stages, including C1, C6, C9, and C10 showed co-expression of *Il17rb* and *Cxcr3* (**Figure 5B**). We thus characterized CD24^-^NK1.1^-^ iNKT cells with different expression statuses of IL-17RB, CXCR3, and CD44. CD138^+^ NKT17 cells were excluded in the gating strategy and PLZF^hi^RORγt^-^ NKT2, PLZF^lo^T-bet^+^ NKT1, CD24^+^CD44^lo^ ST0, NK1.1^+^CD44^hi^ ST3 cells were used in the comparison (**Figures 5C, D**). Different from the mRNA expression data (**Figure 5B**), the flow cytometry analysis showed that 46.64 ± 6.56% and 26.40 ± 2.91% of the cells were IL-17RB^+^ and CXCR3^+^, respectively, while the cells with IL-17RB^+^CXCR3^+^ were only 11.06 ± 0.51% (**Figure 5C**). However, when including more molecules for phenotypic analysis, similarities were found between the subpopulations analyzed by flow cytometry and clusters by scRNA-seq. For instance, P2 (IL-17RB^+^CXCR3^-^CD44^lo/int^) subpopulation corresponded to clusters C0 and C1 in scRNA-seq, ST1 cells (46), or NKTp/NKT2 subsets as the cells in this subpopulation had TCR^hi^PLZF^hi^T-bet^lo^PD-1^hi^CCR7^hi^ phenotype, were larger in cell size with more proliferating cells and high level of glucose uptake (measured by 2-NBDG) (**Figures 5D, E**). By contrast, P4 (IL-17RB^-^CXCR3^+^CD44^hi^) subpopulation was very close to cluster C2 in scRNA-seq or NKT1 subset/ST3 as the cells in this subpopulation exhibited TCR^lo^PLZF^lo^T-bet^hi^CD122^hi^PD-1^lo^CCR7^lo^ phenotype and had smaller cell size, lower level of glucose uptake and proliferation. Compared to P2, P1 (IL-17RB^-^CXCR3^-^CD44^lo^, 4.99 ± 1.01%) subpopulation had a slightly lower level of PLZF, PD-1, CD44 expression, and less glucose uptake, implicating that P1 and P2 subpopulations may correspond to C1 and C0 in scRNA-seq analysis with a precursor-progeny relationship (**Figures 5D, E**). Compared to P4, P3 (IL-17RB^+^CXCR3^int^CD44^int/hi^) and P5 (IL-17RB^-^CXCR3^-^CD44^int/hi^ (P5, 8.92 ± 0.34%) subpopulations showed lower levels of T-bet, CD122, CD44, CD69, higher levels of TCR, PD-1, CCR7, and cell proliferation (**Figures 5D, E**). Thus, the cells in P3 and P5 may correspond to the intermediate precursor clusters such as C3, C4, C6, and C10 in scRNA-seq (**Figures 5C, D**).

As NKT2 cells are the main subset in the thymus of 3-week-old mice while NKT1 cells with ST3 phenotype appear much later (21, 31), we tested the evolvement of these precursor subpopulations with age. In 2-week-old thymi, around 60% of iNKT cells were T-bet^-^PLZF^hi/int^ (NKT2 and NKT17) and 40% were T-bet^+^PLZF^lo^ (NKT1) cells. In 8-week-old thymi, however, the percentages of T-bet^-^PLZF^hi/int^ were less than 20% while T-bet^+^PLZF^lo^ cells reached more than 70% (**Figure 5F**). Similar changes were found in ST1 and ST3 cells in adult mice (**Figure 5F**). The frequency of ST2 cells was first increased in mice at 3-week of age but then decreased with age. In line with the changes of effector iNKT subsets or ST1/ST3 cells, the prevalence of P2 cells showed a substantial decrease while that of P4 cells showed an enrichment as mice aged (**Figure 5F**). No major alterations were found in the relative proportions of P1 and P5 subpopulations while that of P3 showed a first increased then decreased trend with age (**Figure 5F**). Together, these data from C57BL/6 mice suggest that P3/P5 and P4 subpopulations within ST1/ST2 are enriched with intermediate precursors for NK1.1^+^ NKT1 cells.

**Figure 6:**
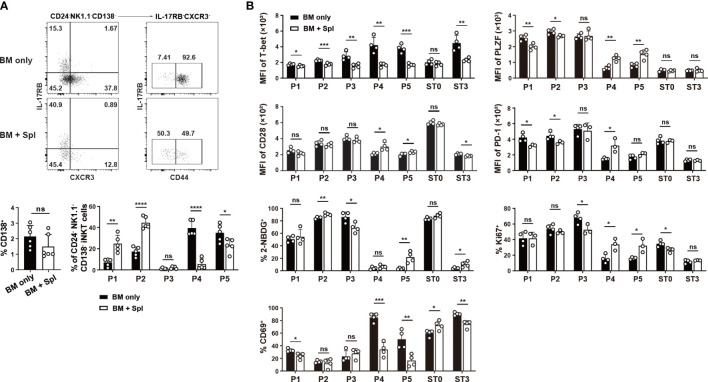
Flow cytometry characterization of reduced thymic NKT1 precursor subpopulations in haplo-BMT mice with GvHD. **(A)** Flow cytometry analysis of the frequencies of CD138^+^ NKT17 subset and P1 to P5 subpopulations. **(B)** Flow cytometry comparison of cell proliferation (Ki67), glucose uptake (2-NDBG), and selected transcription factors and surface molecules in various iNKT subpopulations. *P < 0.05, **P < 0.01, ***P < 0.001, ****P < 0.0001, ns, not significant.

In haplo-BMT mice with GvHD, we observed higher percentages of P1/P2 and substantially lower percentages of P4/P5 subpopulations relative to those with BM only (**Figure 6A**). No difference was found in the relative proportions of CD138^+^ NKT17 subset or P3 subpopulation between the two BMT groups (**Figure 6B**). Notably, the upregulation of T-bet in the few differentiated NKT1 cells (ST3) and their precursor subpopulations (P3, P4, P5) disappeared in GvHD mice (**Figure 6B**). A failure of CD69 upregulation was also observed in P4/P5 subpopulations in GvHD mice (**Figure 6B**). PLZF expression was down-regulated in P4/P5 subpopulations in mice with BM only but was much milder in those with GvHD (**Figure 2B**). We also found higher expression of CD28, PD-1, higher levels of cell proliferation and glucose uptake in P4 and/or P5 subpopulations in GvHD mice (**Figure 6B**). These results suggest that thymic iNKT cells in GvHD mice had impaired NKT1 differentiation at CD44^int/hi^NK1.1^-^ precursor stage with failure to efficiently down-regulate NKT2 signature and upregulate NKT1 signature.

### Enhanced TCR and costimulatory signaling in iNKT cells with GvHD

**Figure 7:**
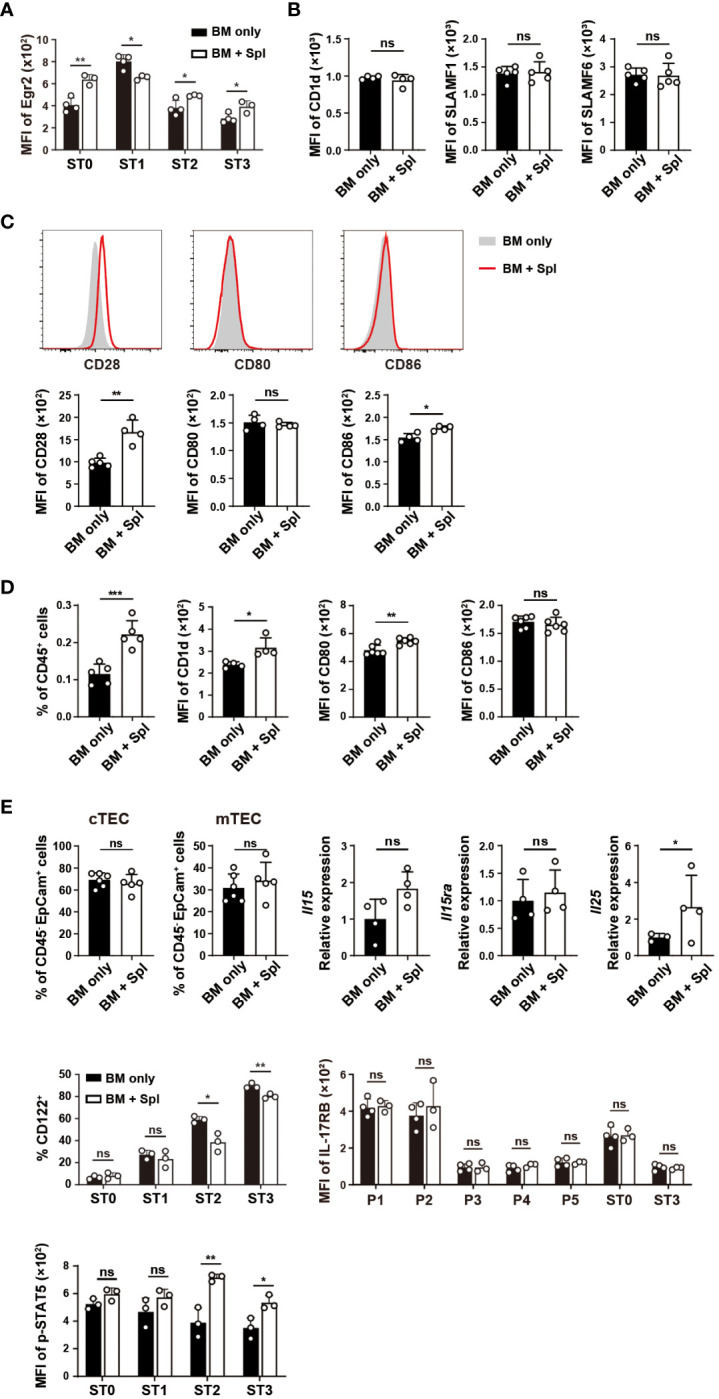
Haplo-BMT mice with GvHD had altered TCR and co-stimulatory signaling in DP thymocytes and macrophages. **(A)** Flow cytometry analysis of Egr2 expression in thymic iNKT cells at various stages. **(B)** Flow cytometry comparison of the expression of CD1d, SLAMF1, SLAMF6 in DP thymocytes. **(C)** Flow cytometry analysis of the levels of CD28 expression and surface-bound CD80 and CD86 in DP thymocytes. **(D)** Flow cytometry comparison of macrophage (CD45^+^CD11b^+^F4/80^+^) frequencies and their expression of CD1d, CD80, and CD86. **(E)** Flow cytometry analysis of the frequencies of cortical and medullary thymic epithelial cells (cTECs and mTECs), expression levels of CD122 (IL-2Rβ), IL-17RB (IL-25 receptor) and phosphorylated STAT5 in iNKT cells, and quantitative PCR analysis of *Il15*, *Il15ra*, and *Il25* transcription in the thymus. *P < 0.05, **P < 0.01, ***P < 0.001, ns, not significant.

The signals from TCR/CD1d, SLAM-SAP, and CD28/CD80/CD86 promote the upregulation of Egr2, PLZF, PD-1, and facilitate the expansion of iNKT cells (20–23, 36, 47). As thymic iNKT cells in GvHD mice also showed increased expression of Egr2 in ST2 and ST3 subsets that contain NKT1 and their precursors (**Figure 7A**), we assessed whether thymic microenvironment that provides TCR and costimulatory signals is altered in GvHD mice. DP thymocytes are critical for the positive selection of iNKT cells and maturation of CD44^hi^ NK1.1^-^ thymocytes (36, 48). Compared to the mice with BM only, those with GvHD showed similar levels of CD1d, SLAMF1, SLAMF6, and surface-bound CD80 (22) in DP thymocytes (**Figures 7B, C**). However, the levels of CD28 expression and surface-bound CD86 in DP thymocytes were significantly higher in GvHD than in BM-only groups (**Figures 7B, C**). Macrophages are the predominant cell type that provides TCR signaling required for NKT2 differentiation (49). We found an increased prevalence of macrophages with upregulated CD1d and CD80 expression in GvHD thymi (**Figure 7D**). These results suggest that GvHD mice have stronger TCR and co-stimulatory signaling in GvHD thymi that may be associated with the increased proliferation, glucose uptake, Egr2, PLZF and PD-1 expression in NK1.1^-^ NKT1 precursors or cells with ST2 phenotype.

Cytokines IL-15 and IL-25 provided by medullary thymic epithelial cells (mTECs) are critical for the differentiation of effector NKT1 and NKT2 cells, respectively (50). As shown in **Figure 7E**, the percentage of mTECs, the expressions of *Il15* and its transpresenting receptor *Il15ra* (51) were comparable between the two groups (**Figure 7E**). The expression of CD122, a component of the receptor for IL-15, was lower in ST2 and ST3 cells in GvHD mice relative to the mice with BM only (**Figure 7E**). The binding of IL-15 receptor to IL-15 leads to the phosphorylation of STAT5 (52). Significantly higher levels of phosphorylated STAT5 were found in ST2 and ST3 cells in GvHD mice (**Figure 7E**). We also determined the transcription of *Il25* in the thymus and found it higher in mice with GvHD. The expression of IL-25 receptor IL-17RB in iNKT cells was comparable between the two groups (**Figure 7E**). These results together suggest that the cytokine signaling for NKT1 and NKT2 differentiation was not impaired in GvHD thymi.

## Discussion

In patients with allo-HSCT, a fast and balanced NKT1 and NKT2 reconstitution is beneficial to reduce the risk of GvHD. GvHD, in turn, is associated with a delay of iNKT cell reconstitution with a more severe impairment of the NKT1 subset. Using the haplo-BMT mouse model, we found a marked reduction of thymic and peripheral iNKT cells with a specific loss of thymic NKT1 subset in mice with GvHD. The reduced number of NKTp cells in the thymus and recent thymic iNKT emigrants in the periphery is likely the main cause for the delayed iNKT reconstitution. We further showed impaired NKT1 differentiation in the thymus of GvHD mice at previously unappreciated CD44^int/hi^CCR7^lo/int^NK1.1^-^ precursor stages.

Mature thymic NK1.1^+^ NKT1 cells (ST3) have been shown to derive from NK1.1^-^NKTp and NKT2-like cells (20, 21, 31–35). NKT1 precursors, however, are not well characterized even though T-bet^+^NK1.1^-^ cells have been detected in CD44^hi^ iNKT cells (29, 36). By excluding NK1.1^+^ ST3 and CD24^+^ ST0 iNKT cells, we were able to enrich and analyze ST1 and ST2 cells at higher resolution. We found three subpopulations of NK1.1^-^ NKT1 precursors (P3-P5) defined by the presence or absence of CXCR3, IL-17RB, and CD44, with gradual down-regulation of NKT2 signature (PLZF, PD-1), upregulation of NKT1 signature (T-bet and CD122), and acquiring tissue persistence features. Together with the late accumulation of the relatively mature subpopulation (P4) with CXCR3^+^IL-17RB^-^CD44^hi^CCR7^-^NK1.1^-^T-bet^hi^ phenotype in young mice, our data suggest that P4 is likely the immediate precursor of NK1.1^+^ NKT1 cells. Both P3 (CXCR3^+^IL-17RB^+^) and P5 (CXCR3^-^IL-17RB^-^CD44^hi^) subpopulations showed transitional phenotype between P2 (NKT2) and P4 and are likely early NKT1 precursors. The relationship between P3 and P5 subpopulations is not clear. This is consistent with the scRNA-seq analysis of NK1.1^-^ iNKT cells where multiple NKT1 precursor clusters (C3, C4, C6, C10) were also found. Based on RNA velocity analysis, the cells in these clusters all have precursors for more mature cluster C2. A clear precursor-progeny relationship, however, was not observed among these early precursor clusters. In addition, relatively more P3 cells were found at steady state while more P5 cells were found in mice receiving BMT or in *Pdcd5* ^-/-^ mice that have defective NKT1 differentiation (data not shown) (53). It suggests that both P3 and P5 iNKT cells may be able to mature into P4 cells. Collectively, the identification of these intermediate NK1.1^-^ precursors of NKT1 cells may facilitate a better understanding of the regulation of NKT1 development.

Lee, Y. J. et al. found that NK1.1^-^ iNKT cells contained terminally differentiated IL-4 producing NKT2 cells (CD4^+^PLZF^hi^CD44^hi^IL-17RB^+^NK1.1^-^), NKT17 cells (CD4^-^CD27^-^CD44^hi^CD122^-^) and the progenitors for NKT1 cells (undefined in NK1.1^-^ cells) (29). They showed that IL-4-producing NKT2 cells could not convert into NKT1 cells in the thymus. In the current study, the PLZF^hi^IL-17RB^+^CD122^-^ subpopulation defined either by scRNA-seq (C0 cluster) or by flow cytometry (P2 subpopulation) had mature NKT2 features including the highest levels of *IL4* transcripts and PD1 expression, and contained no progenitors for other subpopulations (RNA velocity analysis), roughly correlating with the IL-4 producing NKT2 cells reported by Lee (29). We further suggest several CD24^-^NK1.1^-^ subpopulations with NKT1 precursors. At the transcriptome level, the cluster C1 with little *Il4* transcript contained progenitor cells for both NKT2 (C0) and NKT1 (C2) subsets while clusters C3, C4, C6 and C10 had progenitors for NKT1 (C2, RNA velocity analysis). Thus, more direct investigation of precursor-progeny relationship *via* intrathymic injection of subpopulations such as P1 and P3/P5 is needed to validate their differentiation potential.

Antigen presentation and B7-CD28 interaction are essential in many aspects of thymic iNKT cell development, including positive selection before ST0, cell expansion, Egr2/PLZF upregulation, ST0-ST1 transition, and effector NKT subset differentiation and maturation (22, 36, 47, 49, 54, 55). However, how TCR and co-stimulatory signals drive iNKT cell differentiation is not completely clear (21, 25). For instance, mature effector iNKT cells have different levels of TCRs, Nur77, and PLZF that mirror TCR signaling, with NKT1 cells displaying the lowest while NKT2 cells the highest (49). The mice with hypo-morphic ZAP70 allele or Traf3ip3 deficiency showed weakened TCR-MEK/ERK signaling and a decrease in NKT2 but not NKT1 cells (56). In contrast, the mice with Shp1 deficiency that might lead to elevated TCR signaling showed an increase in NKT2 and NKT17 cells (21, 25). Thus, the upregulation of Egr2, PLZF, elevated proliferation, and glucose uptake in NKT1 precursors in GvHD thymi suggest that the strength of TCR and co-stimulatory signaling is higher than normal and may have a negative impact on NKT1 differentiation.

The location (cortex versus medulla) and antigen-presenting cell types that provide TCR and co-stimulatory signaling, the iNKT subsets (ST0/NKTp or intermediate subsets) that receive this signaling to promote effector iNKT differentiation are still under debate (21, 25, 49). The expression of CD1d, CD28, and CD28’s binding of CD80/CD86 on the surface of DP thymocytes have been reported to provide most of the required developmental signals to iNKT cells, including the maturation of NKT1 subset (22, 36). However, how NKT1 precursors recognize CD1d/lipid in the cortex while receiving IL-15 trans-presented by epithelial cells in the medulla is not clear. As a gradual down-regulation of TCR/CD28/CCR7 and upregulation of CD122 were observed in early and late NKT1 precursor subpopulations (CCR7^int/hi^ P3/P5 and CCR7^-^ P4), it is reasonable to think that TCR and cytokine signaling occur sequentially in different precursor subpopulations.

GvHD-induced thymic damage, including the reduction of DP thymocytes, TECs (mTECs in particular), and group 3 innate lymphoid cells, represents one of the major limitations for conventional and unconventional T cell reconstitution following allo-HSCT (13, 57). Alloreactive T cells together with inflammation are responsible for such kind of thymic injury. As the percentages of DP thymocytes and TECs in the current BMT model with GvHD were not reduced, likely due to the small number of splenic cells co-transferred with BM cells, the signals that drive positive selection and cytokines production/presentation required for iNKT differentiation may not be significantly altered. However, we found an increased number of F4/80^+^ macrophages with elevated expression of CD1d/CD80 in GvHD thymi. It has been reported that macrophage activation and differentiation induced by M-CSF, type I IFN, and IL-6 are associated with GvHD development (58). How macrophages affect T cell development under GvHD conditions is less clear. Under the steady-state condition, the macrophages in the medulla are the predominant cells that provide TCR signaling for NKT2 differentiation (49). Thus, the increased thymic macrophages and their elevated CD1d/CD80 expression in GvHD mice may contribute to the preferential differentiation of NKT2 cells and failure of down-regulation of PLZF, PD-1, and cell expansion in NKT1 precursors .

The cause for reduced NKTp and recent thymic iNKT emigrants in GvHD mice is less clear. Reduced proliferation and increased apoptosis at ST0 (**Figure 6A** and data not shown) may contribute to the substantial decrease in the number of NKTp cells. The expression of CD1d and co-stimulatory molecules in DP thymocytes are critical for positive selection, subsequent expansion, and ST0-ST1 transition. Thymic emigration of iNKT cells, however, is independent of TCR signaling (36). In mice with GvHD, the antigen-presenting DP thymocytes showed comparable CD1d/SLAMF1/SLAMF6 expression and elevated CD28/CD86 levels, ST0 iNKT cells displayed increased Egr2 expression, suggesting that TCR/co-stimulatory signaling during positive selection is not altered. Thus, other factors that contribute to the decreased expansion of ST0 cells and subsequent NKTp cells await further investigation.

The percentages of NKT1, NKT2, and NKT17 subsets have been found to be different in mice with different genetic background (59). In B6 mice, the dominant iNKT cell subset in the thymus is NKT1 cells and the percentages of NKT2 and NKT17 cells are few relative to those in BALB/c or FVB/N mice. We thus cannot exclude the possibility that the finding of defective NKT1 differentiation in the thymi of F1 mice with B6-derived donor cells is restricted to the genetic background. Whether NKT2 or even NKT17 cells have defective thymic development in other GvHD mouse models awaits further investigation. However, the TCR signaling strength, duration, and/or kinetics are involved in regulating iNKT subset differentiation in either B6 or BALB/c mice (20–23, 36, 47, 60), with higher signaling strength being necessary for NKT2 and NKT17 subsets development. Thus, the finding of enhanced Egr2 and PLZF expression, elevated proliferation and glucose uptake, indicators of elevated TCR signaling strength in NKT1 precursor cells in GvHD mice support the preferential differentiation of NKT2 subset.

Early recovery of donor-derived iNKT cells in the recipients and/or adoptive transfer of iNKT cells into the recipients are clearly beneficial in reducing GvHD while retaining the GvL effect (2–10, 13–16). However, both human and murine iNKT cells are highly heterogeneous irrespective of the reconstitution *in vivo* or expansion *in vitro* (5, 9, 61). For instance, in human, IL-4-producing CD161^+^CD4^+^ iNKT cells suppressed T cell expansion, CD4^-^CD94^+^ iNKT cells with high cytotoxicity could destroy antigen-presenting cells in the recipients, HLA-II^+^CD161^-^ iNKT cells (mixture of CD4^+^ and CD4^-^ cells) that were more Th1 polarized but less cytotoxic correlated with exhaustion and GvHD (61). In mice, NKT2 and NKT17 cells modulate T cell activation while NKT1 cells have strong anti-tumor activity (10, 59). Whether murine NKT1 cells contain a cytotoxic subpopulation that induces the death of antigen-presenting cells and thus alleviating GvHD is not clear (59). Nevertheless, these results strongly indicate that the compositions of various iNKT cell subsets in addition to cell numbers are critical in promoting a healthy reconstitution in allo-HSCT. Our finding of preferential NKT2 differentiation and delayed NKT1 reconstitution in BMT mice with GvHD may thus inspire more detailed studies of the reconstitution of iNKT subpopulations in relation to various aspects of GvHD and their balance with GvL effect.

Taken together, these data defined NK1.1^-^ NKT1 precursor subpopulations at steady state and identified defects in the differentiation of these NKT1 precursor cells in haplo-BMT with GvHD. Elevated TCR and co-stimulatory signaling in DP thymocytes as well as macrophages may contribute to the failure of down-regulation of the NKT2 signature and upregulation of the NKT1 signature in these NKT1 precursors.

## Materials and methods

### Animals

C57BL/6 (CD45.1 and CD45.2) and BALB/c mice were purchased from Peking University. The animals were kept in a specific pathogen-free facility at Peking University (Beijing, China) and were housed at constant temperature (22-23˚C) with a 12/12h-light/dark cycle and optimal humidity. All animals were allowed to standard lab chow and water ad libitum. The experimental procedures for the use and care of the animals were approved by the Ethics Committee of Peking University (LA2018042). We confirm that all experiments were performed in accordance with relevant guidelines and regulations.

### Mouse model of graft-versus-host disease

F1 (H-2^b/d^, 8-10-week old) mice obtained from the breeding of C57BL/6J (B6, H-2^b^) and BALB/c (H-2^d^) mice were given lethal total body irradiation (10 Gy) as a split dose. T cell-depleted bone marrow cells (5 x 10 (6)) with or without splenic cells (2 x 10 (6)) collected from B6 mice (6-8-week old) were suspended in 200 μl PBS and intravenously injected into F1 recipient mice 4-6 hours after irradiation (62). T cell depletion was performed by magnetic-bead separation using MicroBeads and the autoMACS system (Miltenyi Biotec, Auburn, CA) with negative selection for the CD3 surface antigen. In this model, haplo-BMT recipients with splenic cells developed reproducible GvHD (BM + Spl) as assessed by survival, clinical score while those without splenic cells did not (BM only). GVHD scores were determined by the analysis of recipients’ weight, activity, skin, fur ruffling, and posture as described previously (63).

### Statistics

Statistical analysis was done using GraphPad Prism 8 software (San Diego, CA). Weekly average weight change and GvHD scores were evaluated by two-way analysis of variance (ANOVA) with Sidak’s *post-hoc* comparisons. For all other statistical analysis in this work, we used unpaired or two-tailed paired Student’s *t*-test to evaluate metrics between the study group and the control group. A p value of < 0.05 was considered statistically significant, with more significant values denoted by the number of symbols * < 0.05, ** < 0.01, *** < 0.001, and **** < 0.0001.

Other materials and methods can be found in supplemental materials

## Data availability statement

The datasets presented in this study can be found in online repositories. The names of the repository/repositories and accession number(s) can be found below: GSE228645 (GEO).

## Ethics statement

The experimental procedures for the use and care of the animals were approved by the Ethics Committee of Peking University (LA2018042).

## Author contributions

QG, WZ, YW designed the research, performed research, analyzed data, and wrote the paper. WZ and YW contributed equally. XZ, JH, performed the research, helped with flow cytometry. KZ, HW, RJ contributed animals, critical reagents, and technical supports. XH, YC provided critical suggestions. All the authors reviewed the manuscript. All authors contributed to the article and approved the submitted version.
