# Supplementary material for: Impaired thymic iNKT cell differentiation at early precursor stage in murine haploidentical bone marrow transplantation with GvHD
Source: Front Immunol. 2023 Aug 3;14:1203614. doi: 10.3389/fimmu.2023.1203614 (PMC10438461; doi:10.3389/fimmu.2023.1203614)
Supplement: Supplementary file 1 [file DataSheet_1.docx]

Title: Impaired thymic NKT1 differentiation at early precursor stage in murine haploidentical bone marrow transplantation with chronic GvHD

Weijia Zhao^1,6^, Yujia Wang^1,6^, Xinwei Zhang^1^, Jie Hao^1^, Kunshan Zhang^2^, Xiaojun Huang^3^, Yingjun Chang^3^, Hounan Wu^4^*, Rong Jin^1^*, Qing Ge^1,5^*

**Supplemental materials and methods**

Antibodies and flow cytometry

For iNKT cell analysis, the samples obtained from thymus, spleen, and liver were mechanically disrupted. For stromal cell analysis, thymus tissue was digested with Collagenase D (1 mg/ml, Invitrogen) and DNase I (20 ng/ml, Roche) for 30 min at 37°C. The digestion was stopped by adding EDTA to a final concentration of 5 mM. Single-cell suspensions were prepared and stained with the following monoclonal antibodies purchased from BD Bioscience, BioLegend, or eBioscience: anti-TCRβ (clone H57-597), anti-CD24 (clone M1/69), anti-CD44 (clone IM7), anti-NK1.1 (clone PK136), anti-CXCR3 (clone CXCR3-173), anti-IL-17RB (clone 9B10), anti-Ly-6c (clone HK1.4), anti-Ly-49C/I (clone 14B11), anti-ICOS (clone 7E.17G9), anti-CCR7 (clone 4B12), anti-S1P1 (clone A750 MAB), anti-CD1d (clone 1B1), anti-CD3 (clone 145-2C11), anti-CD4 (clone GK1.5), anti-CD8 (clone 53-6.7), anti-CD122 (clone TM-b1), anti-CD138 (clone 281-2), anti-PD-1 (clone J43), anti-Qa2 (clone 695H1-9-9), anti-CD28 (clone 37.51), anti-CD80 (clone 16/10-A1), anti-CD86 (clone GL-1), anti-CD69 (clone H1.2F3), anti-CD11b (clone M1/70), anti-CD11c (clone N418), anti-F4/80 (clone BM8) and anti-Ly6G (clone 1A8), anti-PLZF (clone Mags.21F7), anti-RORγt (clone B2D), anti-T-bet (clone 4B10), anti-GATA3 (clone TWAJ), anti-Ki67 (clone 16A8), anti-Egr2 (clone Erongr2), anti-Granzyme B (clone GB11), anti-IL-4 (clone 11B11), anti-IFN-γ (clone XMG1.2), anti-pSTAT5 (clone Py694) and anti-pS6 (clone Ser235/236). PE-labeled or APC-labeled CD1d tetramers loaded with or without PBS57 were provided by the Tetramer Core Facility of the US National Institutes of Health. The cell surface staining was performed with 2% fetal bovine serum-containing PBS at 4°C for 30 min. The fixation and permeabilization by Foxp3/Transcription Factor Buffer Set and Intracellular Fixation & Permeabilization Buffer set (eBioscience) were used before staining of transcription factors. All samples were collected on a FACS Canto II Flow Cytometry (BD Biosciences), and data were analyzed with FlowJo software Version 10.6.2.

Activation of iNKT cells.

For *in vitro* activation, splenocytes or liver cells were stimulated with 100 ng/ml PMA (SIGMA), 1 μg/ml ionomycin (SIGMA), and 3 μg/ml BFA (eBioscience) for 5 h at 37°C in complete medium (RPMI 1640, 10% fetal bovine serum). After stimulation, the cells were stained with surface markers and intracellular cytokines. For in vivo activation, each mouse was administered 2 μg α-GalCer (Cayman) by intraveneous injection. Two hours later, the splenocytes and liver cells were isolated and incubated with BFA for 3 h at 37°C before cytokine detection.

Real-time PCR

The thymus tissue was immediately lysed in TRIzol for RNA preparation. cDNA was made using the FastQuant RT kit (TIANGEN Biotech). Real-time qPCR was performed and analyzed as previously described [44]. Expressed levels of target mRNAs were normalized with β-actin and calculated using the 2^–ΔΔCT^ method.

Primer sequences used:

*Il15*: forward, 5’-ACATCCATCTCGTGCTACTTGT-3’

reverse, 5’-GCCTCTGTTTTAGGGAGACCT-3’

*Il25*: forward, 5’-TATGAGTTGGACAGGGACTTGA-3’

reverse, 5’-TGGTAAAGTGGGACGGAGTTG-3’

*Il15ra*: forward, 5’-CCCACAGTTCCAAAATGACGA-3’

reverse, 5’-GCTGCCTTGATTTGATGTACCAG-3’

scRNA-seq library preparation

The thymic single-cell suspension obtained from WT (C57BL/6 mice without BMT), BM only (BMT F1 mice) and cGvHD (BMT F1 mice with BM + Spl) groups was acquired and enriched for CD8^-^ T cells by negative selection using anti-FITC Microbeads and the autoMACS system (Milteny Biotec). The cells with TCRβ^+^CD1d-tetramer^+^CD24^-^NK1.1^-^ phenotype were then sorted by FACS Arial Sorp (BD) and loaded (1,000 cells/µl, cell viability > 90%) onto the Chromium single cell controller (10x Genomics) to generate single-cell gel beads in the emulsion according to the manufacturer’s protocol. The Single Cell 3' Library and Gel Bead Kit (10x Genomics, 1000075), Single Cell 5' Library and Gel Bead Kit (10x Genomics, 1000006), and Single Cell V(D)J Enrichment Kit, Mouse T Cell (1000071) were used and the libraries were eventually sequenced using an Illumina Novaseq 6000 sequencer.

scRNA-seq data processing

The FASTQ sequenced files were aligned with the Mm10 mouse transcriptome using Cell Ranger (v3.1.0). Seurat (v4.0.2) was used to analyze the expression matrix of 3 samples. The following criteria was adopted to control the quality of the data. Cells that expressed > 200 and < 6000 genes and the percentage to mitochondrial gene < 10. A total of 4491, 4201, 2456 high-quality cells with average 32732 genes per cell were obtained from 3 samples for downstream analysis.

Batch correction

The NormalizeData function was used to remove the differences in sequencing depth across cells. The ScaleData function was used to eliminate batch effects caused by batch and mitochondrial gene expression. Then, the harmony (v0.1.0) package was used to reduce the batch effect.

Clustering and Annotation

The RunPCA method was used for dimensionality reduction, and the 1-20 principal components were selected for downstream analysis. Thirteen clusters were obtained at resolution 0.6 by FindNeighbors and FindClusters functions. The RunUMAP function was performed to obtain bidimensional coordinates for clusters. Based on normalized data, differentially expressed genes (DEGs) were identified by FindMarkers or FindAllMarkers function. We further annotated 13 clusters using CellCycleScoring function. Gene Set Enrichment Analysis (GSEA) was performed by using the R package clusterProfiler.

scTCR-seq Analysis

We evaluated the quality of TCR sequenced reads using FastQC, and then aligned the FASTQ sequenced files with the refdata-cellranger-vdj-GRCm38-alts-ensembl-4.0.0 using Cell Ranger VDJ function. Load the filtered_contig_Annotations files of the 3 samples into R, and merge the TCR data of the 3 samples through combineTCR function. Then, we combined the scRNA-seq object with the scTCR-seq data using combineExpression function of scRepertoire (v1.3.4). In order to categorize the frequency of clone types, it was divided into 5 levels [Large (20 < X ≤ 100), Medium (5 < X ≤ 20), Small (1 < X ≤ 5), Single (0 < X ≤ 1), and NA]. Then, we analyzed the differences of TCR clones between clusters and samples.

Pseudotime trajectory inference

Pseudotime trajectory analysis of clusters was generated with Monocle (v2.18.0). The newCellDataSet function (lowerDetectionLimit = 0.5, expressionFamily = negbinomial.size) was used to build the object. Then, we constructed developmental trajectory and visualized dimensionality reduction based on the highly variable genes obtained by the VariableFeatures function, which is identified by Seurat (v4.0.2).

SCENIC analysis

The R packages SCENIC (v1.2.4) were used to infer transcription factor regulatory networks with default parameters. In detail, the co-expression modules were identified through cisTarget databases (mm9-500bp-upstream-7species.mc9nr.feather, mm9-tss-centered-10kb-7species.mc9nr.feather). We first infer regulons on all 11148 cells. Then, the AUCell package (v1.12.0) was used to score the activity of each group of regulons in every single cell.

RNA velocity analysis

Velocyto program was used to perform RNA velocity analysis. We first used velocyto.py to obtain loom files with annotated spliced and unspliced reads of cells. We then performed the following analysis using the velocyto.R pipeline. The gene.relative.velocity.estimates function was used to infer RNA velocity of each cell. The RNA velocity was then projected onto a UMAP dimension reduction diagram, indicating its likely differentiation status by the direction and length of the arrow.

**Supplemental Figure Legend**

Figure S1 Successful establishment of cGvHD and reduced thymic/peripheral iNKT cells in Haplo-BMT mice receiving splenic cells. (A) Comparison of cGvHD score, survival of mice, and body weight between mice with BM only and those with BM + Spl. Two-way ANOVA with Sidak’s post-hoc comparisons was used for statistical analysis. (B) Comparison of the numbers of total thymocytes and the ratios of various thymocyte subsets. The mice with BM + Spl had significantly reduced total thymocytes but the frequencies of DP thymocytes were similar between the two groups. (C) Representative hematoxylin/eosin staining of the thymi obtained from the experimental groups of BM only and BM + Spl after 9 weeks of haplo-BMT. (D) Flow cytometry analysis of iNKT cells in the thymus, spleen, and liver of mice with BM only and those with BM + Spl after 13 weeks of haplo-BMT. (E-F) Flow cytometry analysis of CD44 and NK1.1 expression in iNKT cells in the spleen (E) and liver (F) of mice with BM only and those with BM + Spl after 9 and 13 weeks of haplo-BMT. The percentage and number of CD44^+^NK1.1^-^ and CD44^+^NK1.1^+^ cells were compared. (G) Flow cytometry analysis of donor-derived (CD45.1^+^) and recipient-derived (CD45.1^-^) iNKT cells in the thymus, spleen, and liver 9 weeks after haplo-BMT. (H) Flow cytometry analysis of thymic iNKT cells at various stages in mice with BM only and those with BM + Spl at 13 weeks after haplo-BMT. Data are representative of at least 3 independent experiments. Student’s *t* test was used for statistical analysis. * *P* < 0.05, ** *P* < 0.01, *** *P* < 0.001, ns, not significant.

Figure S2 scRNA-seq analysis of thymic CD1d-tet^+^TCRβ^+^CD24^-^NK1.1^-^ iNKT cells obtained from 8-week-old C57BL/6 mice. (A) PC elbow plot depicting standard deviation of the top 50 principal components. (B) Heatmaps showing expression of 10 most variably expressed genes within clusters C0-C12. (C) UMAP plots with color code displaying indicated gene expression (gray for low and purple for high expression). (D) Violin plots showing cell cycle gene expression changes (reported as normalized G2M and S phase scores) across clusters. (E) Comparison the CDR3 length of TCRβ chain in iNKT cells between BMT mice with BM only and BM + Spl. (F) GSEA analysis of iNKT cell clusters C3, C4, and C6. NES, normalized enrichment score.
