# Supplementary figures and images for: Impaired thymic iNKT cell differentiation at early precursor stage in murine haploidentical bone marrow transplantation with GvHD
Source: Front Immunol. 2023 Aug 3;14:1203614. doi: 10.3389/fimmu.2023.1203614 (PMC10438461; doi:10.3389/fimmu.2023.1203614)

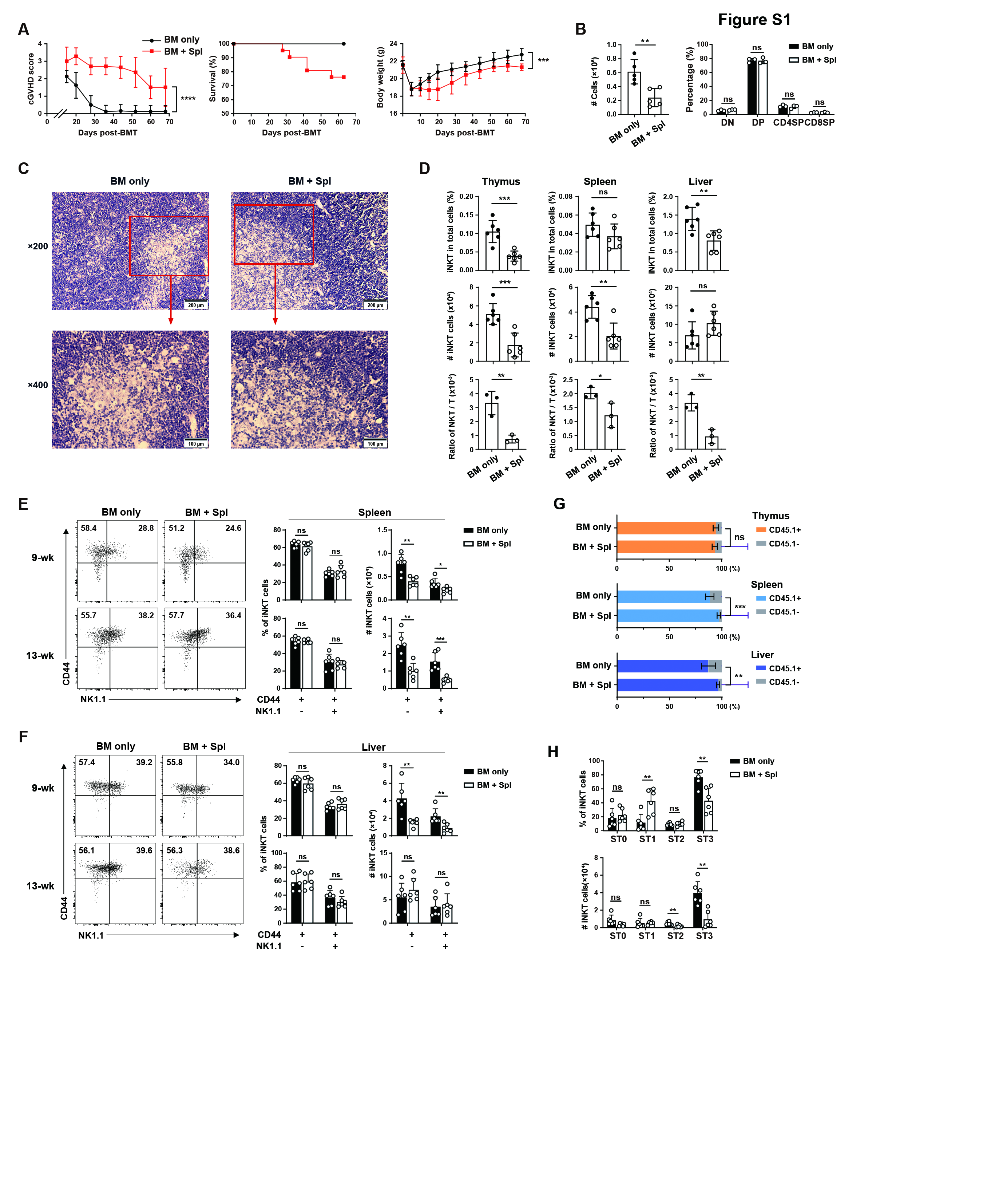

Supplement: Supplementary file 2 [file Image_1.tif]

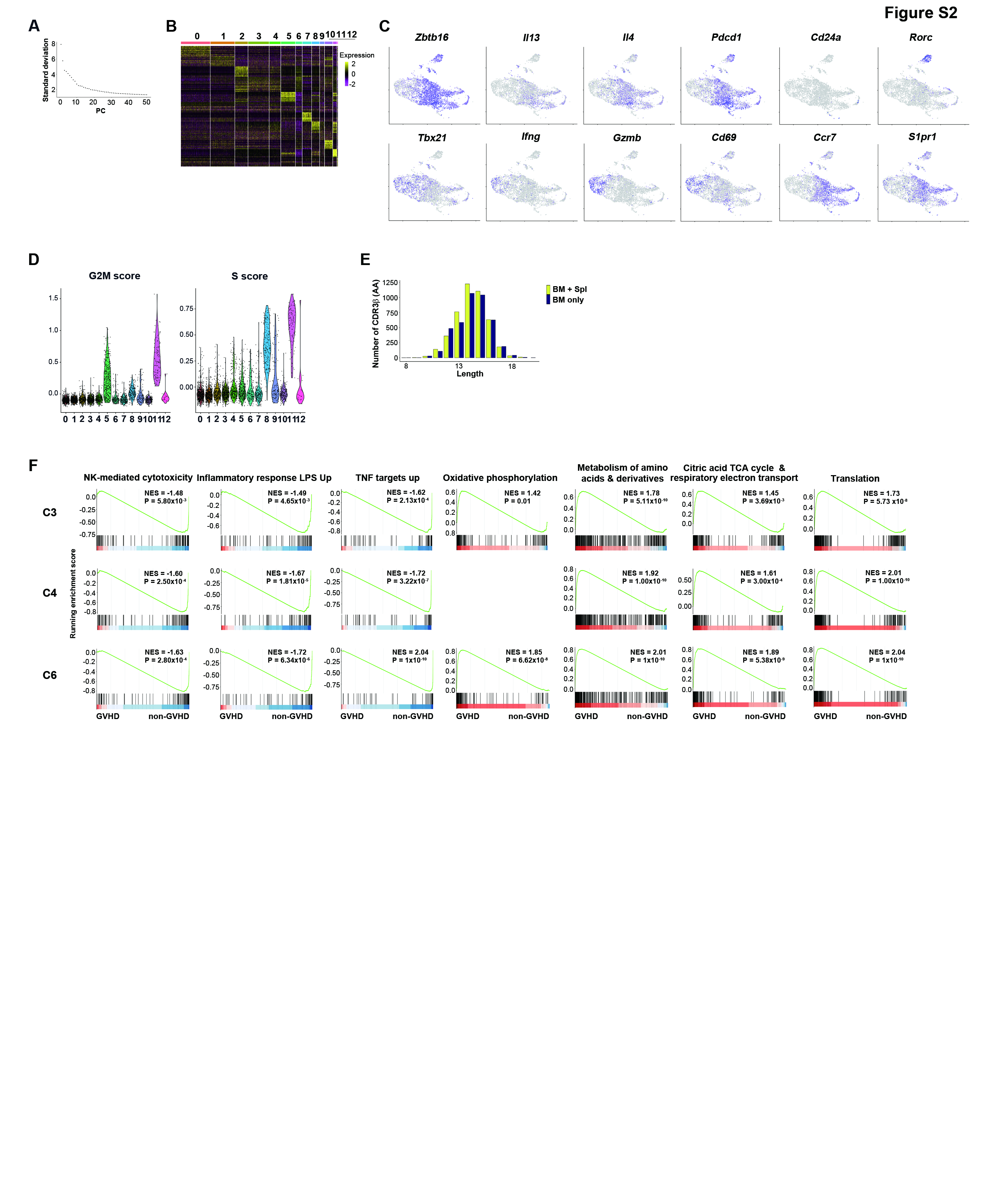

Supplement: Supplementary file 3 [file Image_2.tif]
